# Rodent-specific alternative exons are more frequent in rapidly evolving genes and in paralogs

**DOI:** 10.1186/1471-2148-9-142

**Published:** 2009-06-26

**Authors:** Ramil N Nurtdinov, Andrey A Mironov, Mikhail S Gelfand

**Affiliations:** 1 Departament of Bioengineering and Bioinformatics, M.V. Lomonosov Moscow State University, Vorbyevy Gory 1-73, Moscow, 119992, Russia; 2 State Research Institute for Genetics and Selection of Industrial Microorganisms "GosNIIGenetika", 1st Dorozhny proezd 1, Moscow, 117545, Russia; 3 Institute for Information Transmission Problems, Russian Academy of Sciences, Bolshoi Karenty pereulok 19, Moscow, 127994, Russia

## Abstract

**Background:**

Alternative splicing is an important mechanism for generating functional and evolutionary diversity of proteins in eukaryotes. Here, we studied the frequency and functionality of recently gained, rodent-specific alternative exons.

**Results:**

We projected the data about alternative splicing of mouse genes to the rat, human, and dog genomes, and identified exons conserved in the rat genome, but missing in more distant genomes. We estimated the frequency of rodent-specific exons while controlling for possible residual conservation of spurious exons. The frequency of rodent-specific exons is higher among predominantly skipped exons and exons disrupting the reading frame. Separation of all genes by the rate of sequence evolution and by gene families has demonstrated that rodent-specific cassette exons are more frequent in rapidly evolving genes and in rodent-specific paralogs.

**Conclusion:**

Thus we demonstrated that recently gained exons tend to occur in fast-evolving genes, and their inclusion rate tends to be lower than that of older exons. This agrees with the theory that gain of alternative exons is one of the major mechanisms of gene evolution.

## Background

Alternative splicing is one of the main mechanisms for generating functional and evolutionary diversity of proteins in mammals [1,2]. One of the reasons for that is that new, alternatively spliced exons may introduce a new functionality without sacrificing the old one [2,3]. Initial comparative-genomic analyses of alternative splicing conservation have shown that the fraction of genome-specific alternative splicing may be as large as one fourth to one third of all observed alternatives [2,4,5] whereas recent estimates demonstrate that as much as 93% of human intron containing genes undergo alternative splicing [6,7].

In a study of conservation of human alternatively spliced genes in the mouse genome, we have demonstrated that conservation of cassette exons depends on their expression level (approximated by EST coverage) and their frame-preservation ability [8]. At that, the majority of human-specific cassette exons were singletons and thus could stem from experimental artifacts or errors of the splicing machinery. On the other hand, they still could represent bona fide rare variants that do not have sufficient EST coverage. Indeed, our analysis of EntrezGene and UniGene data demonstrated, that of approximately 29 thousands human genes in EntrezGene ~20% genes have no ESTs at all and further ~20% genes have less than 20 ESTs in UniGene (data not shown).

The human-mouse-dog comparison did not allow us to distinguish between true genome-specific, recently gained instances of alternative splicing and errors and artifacts. Similarly, while the human-mouse-rat comparisons that has demonstrated that ~60% of cassette exons conserved in mouse and rat are not conserved in human and ~20% of cassette exons conserved in human and one rodent are not conserved in the other [9] are sufficient for the estimation of the loss rate of cassete exons; they do not allow one to estimate the rate of the cassette exon gain.

A mouse-rat-human comparison with pig as an outgroup was used to estimate the rate of exon birth in rodents [10] (new exons were defined as exons conserved in mouse and rat, and missing in the human genome and pig ESTs). The majority of rodent-specific exons were alternative. While this is a definite step forward compared to the triple comparisons, there still are two problems with this approach. Firstly, EST coverage of the pig genome may be not sufficient to guarantee that an exon missing in the EST data indeed is not present in the genome. This is especially true for young exons, rarely included in the mature mRNA. Secondly, the mouse-rat conservation alone may not be sufficient to claim the functionality. Indeed, mouse exons that could not be aligned to the rat genome were not considered at all, and thus some conservation is expected simply by definition: in a conserved region in DNA, chance activation of cryptic sites would create a seemingly conserved exon. Both these possibilities would yield over-estimation of the number of functional rodent-specific exons.

One way to address this issue is to use additional genomes in order to consider not genome-specific, but lineage-specific alternatives. This was done in [11] where eight completely sequenced vertebrate genomes were considered and in [12], where human genes were compared to the ENCODE genome fragments from seventeen vertebrates. Both studies demonstrated that the fraction of cassette exons, especially minor isoform ones, is larger in the cohort of young (lineage-specific) exons. However, these studies did not control for functionality of these exons.

Here we analyzed mouse genes in the same mouse-human-dog triples as in our previous study [8], but additionally considered conservation of mouse exons in the rat genome. At that, we also created a control sample of mouse pseudoexons, that is, intron regions bounded by potential splice sites, and subjected it to the standard procedure for estimating conservation. This allowed us to estimate the frequency of bona fide rodent-specific exons.

One additional, interesting question is the possible correlation between the rate of evolution of alternative splicing and the rate of protein sequence evolution. Such analysis should be performed carefully, to avoid the ascertainment bias. Indeed, while the observed frequency of alternative splicing increases with the EST coverage of genes [13], highly expressed genes tend to evolve slowly [14,15]. The observation that slowly evolving genes tend to be more frequently alternatively spliced compared to moderately and rapidly evolving genes has been made in [16].

While it is natural to expect that rapidly evolving genes also have rapidly evolving alternative splicing, a significant fraction of observed alternative variants may represent splicing errors. Hence it is necessary to take into account the frequency of an alternative variant and its frame preservation properties. When only genes with frame-preserving frequently inserted alternative exons were considered, it turned out that indeed constitutive regions of genes with human-specific exons evolved faster than similar regions of genes with conserved exons [16]. However, genome-specific cassette exons considered in this study still could be non-functional. Here, we address this problem by the analysis of exon conservation in two rodent genomes. Further, this study did not estimate the rate of alternative exon gain and loss.

Finally, we address the question of emerging alternative splicing in paralogs. Previous studies have demonstrated that duplicated genes are less frequently alternatively spliced than singletons [17,18]. To survive, duplicated genes need to gain new functionality that, in particular, can be introduced by new exons. We analyzed the rate of exon emergence in paralogs and their non-duplicated orthologs.

## Results and Discussion

Orthologous human, mouse, dog and rat genes were taken from Homologene [19]. Clusters where each gene contains at least one intron in the protein-coding region in all four genomes were selected. All quartets of orthologous genes were assigned to three groups according to the similarity level of coding proteins. To avoid the influence of non-alignable genomes-specific exons, for this analysis we considered only orthologous exons. Several papers have showed that alternatively spliced regions evolve more rapidly compared to the constitutively spliced ones [[20,21], reviewed in [3]], but it substantially affects only minor isoform cassette exons [22]. The numer and total length of such exons is low compared to constitutive, and they should not strongly influence average similarity level. We defined 2693 rapidly evolving genes that had the similarity between 0.4 and 0.8, while the remaining 7386 genes were split in almost equal parts of 3939 genes with similarity between 0.8 and 0.92 and 3447 genes with similarity exceeding 0.92.

To define duplicated genes, we initially determined the best human hit for each mouse gene, and formed families of mouse genes that shared the human ortholog. Then we identified the rat ortholog for each mouse gene. We retained only those members of the families, that had rat orthologs, indicating that they had duplicated prior to the mouse-rat divergence (see the "Methods" for details). This resulted in 110 rodent-level duplication families consisting of 269 genes.

Alternative splicing of mouse genes was analyzed by aligning all available sequences and analysis of the splicing graphs as in [8]. All cassette exons were divided in two groups, frame preserving and frame-disrupting ones. The latter group consisted of frame-shifting exons or exons containing in-frame stop-codons. For each cassette exon, we calculated its inclusion ratio defined as the fraction of the number of sequences fragments containing this exon to the total number of fragments covering the corresponding gene region. Rare exons that potentially could arise from splicing errors were defined using the procedure from [23], see Methods.

The mouse and rat lineages diverged about 16 million years ago [24]. Thus, as mentioned in the Introduction, one has to control for residual conservation in regions containing spurious, non-functional exons. We assumed that conservation of mouse cassette exon in the human or dog genomes is sufficient to interpret them as real exons. The remaining (candidate) exons could be conserved in the rat genome either spuriously or because of functional importance. We created a set of randomly selected mouse pseudoexons (random regions of introns with the same length distribution, bounded by canonical AG-GT dinucleotides) and tested their conservation in the rat genome using exactly the same procedure as the one applied to real exons. The average conservation of pseudoexons depended on their length and usually belonged to the interval (0.05; 0.15). Thus testing the conservation of 100 mouse candidate exons in the rat genome we should expect that 5 to 15 of these exons could be conserved spuriously.

To take this into account, for each mouse cassette exon not conserved in the human or dog genomes, we considered all pseudoexons with same length and calculated their residual conservation probability. The sum of these probabilities over all candidate exons provided an estimate for the number of spuriously conserved rodent-specific cassette exons. Thus the estimated number of exons conserved because they are functional is the total number of observed conserved exons minus this value.

**Figure 1:**
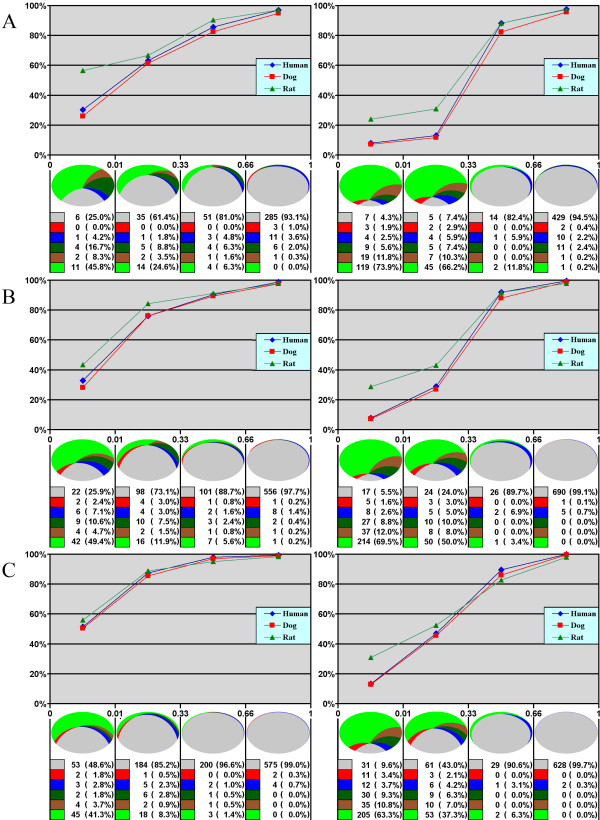
**Conservation of mouse frame-preserving (left) and frame-disrupting (right) cassette exons with different inclusion level in human, dog and rat genomes for different groups of genes: (A, B, C) rapidly, moderately, and slowly evolving genes, respectively**. Top plots: blue diamonds, red squares and green triangles – the fraction of mouse cassette exons conserved in the human, dog and rat genomes, respectively. Crescent pie charts below: the segment sizes are proportional to the number of exons conserved in the human, dog and rat genomes (grey), exons conserved in the rat genome and in the human or dog genome but not both (blue and red, respectively), exons conserved only in the rat genome (dark green for estimated functional conservation, brown for residual conservation, see the text for details), mouse-specific exons (green).

The levels of conservation of mouse frame-preserving and frame-disrupting cassette exons in the human, dog and rat genomes orthologs with different rate of sequence evolution are analyzed in Figure 1. The conservation of mouse cassette exons noticeably depends on their inclusion level and frame preservation, with predominantly included and frame-preserving exons being more conserved than predominantly skipped and frame-disrupting exons, respectively.

While we cannot tell whether a particular rodent-specific exons is functional, we used the procedure described above to estimate the number of real rodent-specific exons, and it is non-negligible in all groups. The fraction of evolutionary young, rodent-specific cassette exons falls as the exon inclusion level increases, in agreement with the theory that new exons emerge as rarely included cassette exons [2,3,25]. This fraction is higher in rapidly evolving genes and lower in slowly evolving ones, thus demonstrating the correlation between the two modes of gene evolution.

We observed 107 cassette exons in duplicated genes. To compare the birth rates of rodent-specific cassette exons, we summarized the data for the duplicated genes, and compared it with the information about orthologous genes (Table 1).

**Table 1:** Summary information about conservation of mouse cassette exons

	**rodent duplications**	**rapidly evolving orthologous genes**	**moderately evolving orthologous genes**	**slowly evolving orthologous genes**	**all orthologs**
**Conserved exons**	72 (67.3%)	877 (76.3%)	1591 (78.1%)	1813 (80.9%)	4281 (78.9%)
**Rodent-specific exons**	18 (16.8%)	77 (6.7%)	114 (5.6%)	100 (4.5%)	291 (5.4%)
**Estimated real rodent-specific exons**	10 (9.3%)	44 (3.8%)	61 (3.0%)	48 (2.1%)	153 (2.8%)
**Mouse-specific exons**	17 (15.9%)	196 (17.0%)	331 (16.3%)	327 (14.6%)	854 (15.7%)

**All cassette exons**	107	1150	2036	2240	5426
**Genes**	269	2693	3939	3447	10079
**Cassette exons per gene**	0.40	0.43	0.52	0.65	0.54

The frequency of mouse-specific exons is the same in all groups of genes, and this may be explained by the fact that most of these exons are not real and are due to experimental artifacts or splicing errors. We confirmed lower frequency of alternative splicing in duplicated genes compared to non-duplicated ones. We also observed that the frequency of genes with cassette exons decreases from slow to rapidly evolving genes. On the other hand, the frequency of rodent-specific exons was higher in duplicated genes compared to non-duplicated ones, and it increased from slow to rapidly evolving genes. This is consistent with the observations about the rate of exon birth in different groups of genes. Genes with faster molecular evolutionary rate are more likely to gain a new, alternatively spliced exon.

## Conclusion

A popular theory [2-4,25-27] posits that alternative splicing is one of the main mechanisms of increasing protein diversity in eukaryotes. At that, exonisation of intronic regions creates alternative exons that may subsequently become constitutive by fine-tuning of splicing regulatory sites. At the same time, the new protein fragment evolves under positive selection [22].

Our observations are consistent with the predictions of this theory. Indeed, we have demonstrated that recently gained, rodent-specific exons are more prevalent in relatively fast-evolving genes and in faster evolving paralogs in rodent-specific duplicated genes. We have demonstrated further, that recently gained exons are incorporated into a minority of mature mRNA isoforms.

## Methods

The initial sample of 12622 human, mouse, dog and rat orthologous genes was taken from Homologene [28] and EntrezGene [29]. We used NCBI Build 36.1 version of the human genome, NCBI Build 37 version of the mouse genome, RGSC v3.4 version of the rat genome and the May 2005 dog (*Canis familiaris*) whole-genome shotgun (WGS) assembly v2.0. 11963 clusters where each gene contains at least one intron in the protein-coding region in all four genomes were selected. Exon-intron structure for each gene was reconstituted by aligning corresponding proteins from Homologene to genome sequences. To evaluate the molecular evolution rates we compared protein sequence of orthologous exons and measured the similarity using the Blosum62 matrix. We used only human, mouse and dog exons, molecular evolution rate of rat genes was assumed to be the same as for the mouse genes. Orthologous exons with similarity less than 0.25 were filtered out. Further, 911 gene clusters were filtered out because their orthologous exons covered less than 75% of initial protein-coding sequences in human and mouse.

To define duplicated genes, we used Blat [30] to align protein sequences of 21791 intron-containing mouse genes with protein sequences of 19718 human genes from EntrezGene [29]. For each mouse gene the best human hit was selected. Mouse genes aligned to the same human gene were considered to be candidate inparalogs and formed duplication families. We verified that these genes were more similar to each other than to the orthologous human gene. This resulted in 250 families consisting of 637 genes. Further we defined rodent-specific inparalogs, i.e. genes that duplicated before the divergence of mouse and rat lineages but after the divergence of rodents and primates. For each member of mouse families we searched Homologene [28] for the orthologous rat gene, the absence of such ortholog for the particular genes indicating that the duplication had occurred in the mouse linage after the divergence of rat and mouse ancestors, or the gene had not been sequenced in the rat genome. These genes were filtered out. This resulted in 110 rodent-level duplication families consisting of 269 genes.

All protein, mRNA, DNA and EST sequences were derived from GeneBank [19] (UniGene, EntrezGene, GenePept). EST and mRNA sequences were aligned with genomic DNA using ProEST [31], and protein sequences were aligned with genomic DNA using ProFrame [32]. For each gene we constructed the splicing graph and defined cassette exons. Rare exons and exon-skipping events that could arise from splicing errors were defined using the procedure from [23]. Briefly, a variant was considered "rare" (and hence suspicious), if the hypothesis that its frequency is less than 1% could not be rejected at 95% significance level given the observed counts of variants of the considered cassette exon, see [8] for details.

Conservation of cassette exons was assessed by the analysis of DNA to DNA alignments of orthologous genes. At the first step, an alignment was split into intervals between well conserved exons defined by Blat [29], and then we attempted to identify the remaining exons by genomic spliced alignment using ProGene [33], see [8] for details.

We created a set of randomly selected mouse pseudoexons in constitutively spliced introns, 10799 exons with and 14448 exons without termination codons, and tested their conservation in the rat genome using exactly the same procedure. Conservation of these pseudoexons depends on their length and presence or absence of internal stop-codon and we fitted the sample size to achieve coverage of more than 100 pseudoexons per each 12-nucleotides interval of exon lengths.

## Authors' contributions

MSG and AAM conceived the study. RNN performed the analysis. RNN, AMM and MSG analyzed the results. RNM and MSG wrote the manuscript. All authors read and approved the final manuscript.
